# Acorn cotyledons are larger than their seedlings' need: evidence from artificial cutting experiments

**DOI:** 10.1038/srep08112

**Published:** 2015-01-29

**Authors:** Xianfeng Yi, Zhenyu Wang, Changqu Liu, Guoqiang Liu, Mingming Zhang

**Affiliations:** 1 College of Life Sciences, Jiangxi Normal University, Nanchang 330022, China; 2 College of Agriculture, Henan University of Science and Technology, Luoyang, 471003, China

## Abstract

Although the consequences of cotyledon removal have been widely studied in oaks producing large acorns, we have little knowledge of at what level cotyledons can be removed without affecting acorn survival and seedling development. In this study, we aimed to test the hypothesis that the amount of energy reserves in cotyledons is more than the demands of seedlings and that large acorns can tolerate seed predation and/or attract seed predators for seed dispersal. Acorn germination rates were not affected even when 60% of cotyledons were cut off at the basal end, suggesting that the energy reserves contained in cotyledons are not essential for acorn survival. Post-cut acorn mass, more than initial acorn mass, appear to be a better predictor of seedling performance, indicating that the energy reserves in cotyledons are sufficient for seedlings. Acorns with large masses sustained cotyledon damage better than small ones with respect to seedling performance. Large acorns were more likely to be dispersed and cached by animals, implying that producing large acorns is more important for oaks to manipulate seed predators and dispersers rather than provide a seedling with cotyledonary reserves.

Seed size plays an important role in determining seed dispersal, seed survival, and seedling establishment, and has been widely studied in the life history stage of plants^1^. A large number of animals are necessary for seed dispersal of various plant species producing propagules (e.g., seeds or fruit) in a wide range of sizes^2^,^3^,^4^,^5^. Although large seeds have been found to produce large seedlings with higher fitness and performance estimates^6^,^7^,^8^,^9^,^10^, they are more vulnerable to be damaged during seed dispersal processes compared to those with small sizes, due to their attractiveness to seed predators^11^,^12^.

Damage to seeds has been found to cause decreases in germination rates, reduction of seedling performance, and even death of seeds or seedlings^13^,^14^. Fukumoto and Kajimura^15^ show that survival of hypocotyls and radicles of *Quercus variabilis* is severely affected by high-level cotyledon loss. Kennedy et al.^16^ found that seed reserves have an important effect on the early performance of *Lithocarpus densiflora* seedlings. Cotyledon removal just after the emergence of seedlings poses significant negative impacts on seedling growth of oaks^17^. Significant consequences of cotyledon extirpation of seedling growth in oaks have been widely observed in previous studies^18^,^19^,^20^. Existing literature indicates that energy reserves in cotyledons play an important role in supporting seedling development; therefore, damage to seeds can negatively influence seed survival or seedling development.

On the other hand, large reserves in seeds act as a potential nutrition pool and help seedlings overcome the negative effect of cotyledon damage by seed predators^6^,^7^,^21^,^22^,^23^. An increasing body of literature indicates that partially consumed acorns can germinate and successfully develop into normal seedlings provided that the vulnerable embryonic parts are not damaged by animals^14^,^23^,^24^,^25^,^26^,^27^,^28^. Previous studies show that cotyledon damage in acorns of various oak species usually causes an insignificant impact on seedling performance^23^,^25^,^29^,^30^,^31^ and may even increase germination rates in some species^25^,^32^. For instance, Dalling and Harms^33^ showed that the large-seeded tropical species *Gustavia superba* can tolerate severe cotyledon reduction (even up to 50%) to survive. Similar studies in several oak species found that large acorns tolerate insect predation better than small ones^20^,^23^. Acorns of *Q. robur* can still germinate and successfully establish seedlings with a loss of up to 2/3 their cotyledon biomass^32^. Recent studies showed that white oak radicles >6 cm containing the plumule can successfully regenerate into seedlings even without the nutrition supplied by cotyledons^28^,^34^. These findings imply that the cotyledonary reserves in acorns may be not crucial for supporting seedling development and acorns may serve as food for manipulating seed predators and dispersers. Although the consequences of cotyledon loss have been widely studied in the early development of oak seedlings^17^,^32^, it remains unclear how much of the cotyledonary reserve can be removed without affecting acorn survival and seedling development.

To test the hypothesis that large acorns act as resource reserves to tolerate seed predation and/or to attract seed predators for seed dispersal, we determined the consequences of successional cutting off the distal (basal) end of acorns on the development of *Quercus mongolica* seedlings. We also released paired large and small acorns of *Q. mongolica* in the field to track seed dispersal and seed survival, as well as seedling establishment. We predicted that: 1) there would be a threshold of cotyledon loss that significantly reduced the germination rate and seedling performance; 2) acorns with large masses would be more able to sustain cotyledon loss compared with small-sized acorns, especially when a large proportion of cotyledon was lost; 3) seedling performance would be closely correlated with post-cut acorn mass rather than the initial one; 4) large acorns would have greater chances to be dispersed and scatter-hoarded by animals, and consequently show higher survival rates.

## Methods

### Plant material

Mature acorns of *Q. mongolica* were collected in Dongfanghong Forest Park (128°57′16″–129°17′50″E, 46°50′8″–46°59′20″N) in a seed masting year 2012. Acorns were stored in a cool room at 4°C and 60% of humidity. After 10-month storage, 750 acorns were randomly selected and individually peeled. Each peeled acorn was weighed for initial mass and then assigned to 15 experimental treatments, each with 50 acorns. Fifteen distinct damage classes were then created by removing cotyledon biomass of these acorns from 0% to 70% of removal (with a 5% of interval from one level to another). We removed cotyledons in this manner because acorns are more likely to be damaged by insects and vertebrates at the distal end^27^,^35^,^36^. Each acorn was weighed after cutting in order to control the exact proportion of cotyledon loss. Then, these acorns were sown vertically at 1–2 cm in depth individually in plastic containers (15 cm in radius and 20 cm in height) filled with soil collected in the forest. Each of the 15 treatments had 5 containers, in which 10 acorns were sown evenly. Each acorn was given a unique ID by inserting a numbered toothpick just beside the acorn, making it easy to identify the corresponding seedling after germination. All containers were placed 30 cm apart under natural conditions in an enclosure to prevent seed predation and watered regularly as necessary. The experiments were carried out in the Field Experimental Base of the Institute of Zoology, CAS (mean elevation of 750 m, 45°58′N, 129°08′E) in Dailing District, Yichun City, Heilongjiang Province, northeastern China.

### Growth parameters

The germination of acorns and the survival of seedlings throughout the growing period were recorded. Germination rates were measured every day after sowing for 50 days. Seedling height and leaf number of each seedling were measured at the end of the growing season (i.e., at the end of August). Each seedling was cleaned with distilled water and oven-dried (70°C for 48 h) so as to obtain a measurement of the dry masses of roots and shoots (i.e., epicotyl and leaves). The roots and shoots of each seedling were weighed separately to the nearest ±0.01 g. Dry masses of roots and shoots were summed up to detect correlation of the total seedling biomass with acorn mass (initial or post cut), percent acorn damage, and the amount of cotyledon loss.

### Acorn dispersal by animals

*Q. mongolica* acorns were collected from 10–15 oak trees during the period of seed fall in the study area. We released 10 paired large and small acorns (4.04 ± 0.36 g vs 2.72 ± 0.02 g, mean ± SE) into each of the 66 seed stations during seed fall in 2010. Totally, 660 large and 660 small acorns were tagged and released. After seed release, we searched the area around each seed station (radius, <30 m) every day for 5 days to determine seed fates. Seed fates were sorted into six categories: 1) intact in situ (IIS); 2) eaten in situ (EIS); 3) eaten after removal (EAR), 4) intact after removal (on surface) (IAR); 5) scatter-hoarded after removal (in soil or litter) (SH); 6) missing (M). The next spring, large and small acorns in scatter-hoards were checked to see whether they successfully germinated into seedlings.

### Statistical analyses

We used Statistical Package for the Social Sciences (SPSS 16.0) for data analysis. ANOVA was used to determine if there were differences in the initial (post-peeling mass) and post-cut acorn mass between the control and treatment groups. One-sample Kolmogorov-Smirnov test was used to determine whether the dependent variable was normally distributed. Linear regression was used to determine if initial mass (independent variable) of an individual acorn predicted the final total biomass (dependent variable) of the resulting seedling using only control acorns. Linear regression was also used to determine the effects of post-cut acorn mass and percent acorn damage (dependent variable) on germination rates and seedling performance (dependent variable). Linear regression was also used to determine the correlation between seedling dry masses (dependent variable) and exact cotyledon loss amounts (independent variable) in all treatment groups, to see if large acorns tolerated cotyledon damage better than small ones. Cox regression was used to test the difference in acorn removal rates between large and small acorns following arc-sine transformation. Paired samples *t*-test, following arc-sine transformation, was used to see if there was a difference in the proportion of large and small acorns eaten or scatter-hoarded by animals.

## Results

### Acorn characteristics after artificial damage

*Q. mongolica* produced acorns with masses ranged from 1.71 to 6.40 g (mean ± SD: 3.18 ± 0.70 g), exhibiting a 4-fold difference between the largest and smallest acorns. The average initial acorn masses of the 15 groups were 3.57 g, 3.62 g, 3.53 g, 3.37 g, 3.14 g, 3.03 g, 2.89 g, 3.01 g, 2.82 g, 3.06 g, 3.07 g, 3.14 g, 3.09 g, 3.25 g, and 3.12 g, respectively. After delicate successional cutting, we removed 0%, 5.15%, 10.40%, 14.63%, 19.79%, 22.04%, 30.61%, 35.39%, 39.43%, 44.73%, 49.35%, 54.83%, 59.02%, 64.63%, and 69.13% of the cotyledon masses of the 15 groups, respectively. The average post-cut cotyledon masses of the 15 treatments were 3.57 g, 3.44 g, 3.17 g, 2.87 g, 2.51 g, 2.37 g, 2.00 g, 1.94 g, 1.70 g, 1.69 g, 1.54 g, 1.42 g, 1.26 g, 1.14 g, and 0.96 g, and differed significantly (F_14, 735_ = 172.453, P < 0.001). After artificial damage, the final cotyledon masses of the 15 treatments ranged from 0.50 to 5.07 g (mean ± SD: 2.11 g ± 0.93), 33.6% less than the initial acorn masses.

### Seedling performance

**Figure 1:**
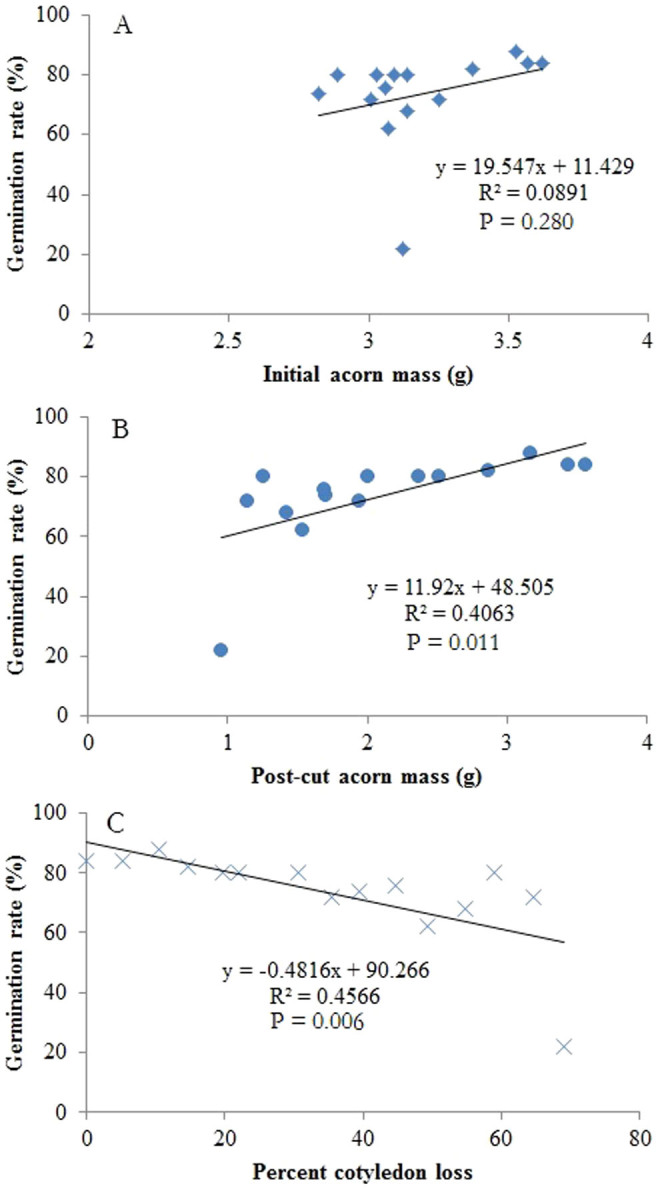
Effects of initial acorn mass (A), post-cut acorn mass (B), and percent cotyledon loss (C) on germination rates of *Q. mongolica* acorns.

**Figure 2:**
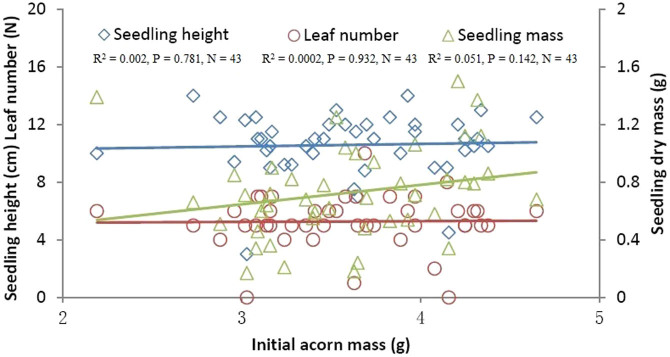
The effect of initial acorn mass on seedling height, leaf number and dry mass of *Q. mongolica* seedlings. The points in the figure show the original data.

**Figure 3:**
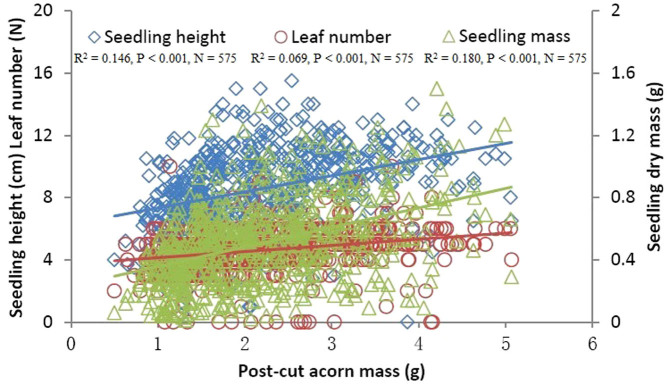
The effect of post-cut acorn mass on seedling height, leaf number and dry mass of *Q. mongolica* seedlings. The points in the figure show the original data.

**Figure 4:**
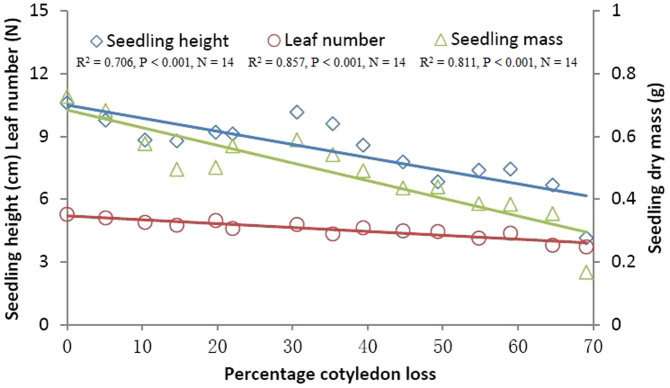
The effect of percent cotyledon loss on seedling height, leaf number and dry mass of *Q. mongolica* seedlings. The points in the figure show the means.

Cotyledon-removed acorns that germinated all survived in each damage class in our study (Fig. 1). No correlation was found between initial acorn masses and germination rates (Fig. 1A), whereas, germination rates were significantly positively correlated with post-cut acorn masses (Fig. 1B). Although germination rates were significantly correlated with percent cotyledon loss (Fig. 1C), cotyledon loss less than 70% caused no significant effect on germination rates of damaged acorns (F_13, 56_ = 0.711, P = 0.744). However, treatment of 70% cotyledon loss resulted in an extremely low germination rate compared with the control group (F_1, 8_ = 17.603, P < 0.001). These facts suggest that damage to cotyledons exerts a minor impact on acorn survival. Using control acorns, seedling performance (seedling height, leaf number, and total final biomass of seedlings) was not predicted by initial acorn mass (Table 1, Fig. 2). However, post-cut acorn mass appeared to be an important determinant of seedling height, leaf number, and total final biomass (Table 1, Fig. 3). Moreover, we found significant effects of percent acorn damage on seedling height, leaf number, and total final biomass (Table 1, Fig. 4). In cutting treatments with the percent cotyledon loss less than 60%, seedling dry masses were not correlated with initial acorn masses (5%: R^2^ = 0.001, P = 0.885; 10%: R^2^ = 0.003, P = 0.713; 15%: R^2^ = 0.007, P = 0.600; 20%: R^2^ = 0.041, P = 0.210; 25%: R^2^ = 0.021, P = 0.368; 30%: R^2^ = 0.126, P = 0.023; 35%: R^2^ = 0.017, P = 0.431; 40%: R^2^ = 0.004, P = 0.696; 45%: R^2^ = 0.189, P = 0.005; 50%: R^2^ = 0.009, P = 0.600; 55%: R^2^ = 0.016, P = 0.495). However, seedling dry masses were positively correlated with initial acorn masses for cutting treatments with percent cotyledon loss of 60% and 65% (60%: R^2^ = 0.256, P = 0.001; 65%: R^2^ = 0.136, P = 0.021). These results indicate that large acorns tolerate cotyledon damage better than small ones at a higher level of percent cotyledon loss.

### Acorn dispersal by animals and seedling establishment

**Figure 5:**
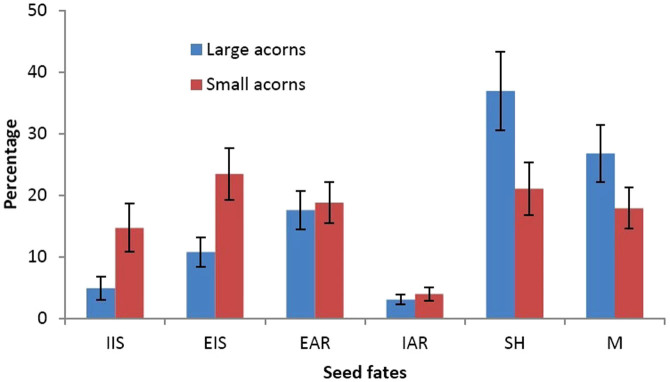
Seed fates of large and small acorns of *Q. mongolica* manipulated by small rodents in the field. Data are expressed as mean ± SD. IIS, EIS, EAR, IAR, SH, and M stand for seeds intact in situ, eaten in situ, eaten after removal, intact after removal, scatter-hoarded after removal, and missing, respectively.

Our field survey demonstrated that 95.2% and 85.3% of large and small acorns were harvested by animals 5 days after seed placement, respectively. Seed removal rates differed significantly between large and small acorns (Wald = 5.340, df = 1, P = 0.021). Small acorns were more likely to be eaten in situ than large acorns (t = −4.681, df = 65, P < 0.001) (Fig. 5). However, large acorns were more likely to be scatter-hoarded compared to small ones (t = 5.894, df = 65, P < 0.001) (Fig. 5). In the following spring, we relocated 14 seedlings from large acorns while 6 from small acorns, showing significant difference (Chi-square test: χ^2^ = 5.556, df = 1, P = 0.018).

## Discussion

### Effect of cotyledon removal on acorn survival

Previous investigations usually evaluate the consequence of cotyledon damage at 1–3 different levels of artificial cotyledon removal^14^,^17^,^24^,^26^,^32^. In our study, successive cutting experiments showed that acorn germination rates were not affected even when 65% of cotyledons were cut off at the basal end. However, the survival rate of acorns decreased dramatically with a loss of over 70% of cotyledon biomass. This is expected to be the first artificial cutting study showing the maximum capacity of oak acorns to sustain cotyledon removal or damage. Our results further support previous studies that show that acorns can successfully establish seedlings even after losing up to 50% of their cotyledons^32^. These results imply that the amount of energy reserves in oak cotyledons is much more than the demands of young seedlings^32^.

### Effect of cotyledon removal on seedling performance

Our data showed that post-cut acorn mass, rather than initial mass, appeared to be a better predictor of seedling performance (e.g., seedling height, leaf number, seedling dry mass). At lower levels of cotyledon damage (<60%), large acorns showed no advantage over small ones with respect to seedling dry mass. However, seedlings from large acorns appeared to perform better than those from small ones at higher levels of cotyledon damage (i.e., 60% and 65%), partially supporting our prediction that large acorns sustain damage to cotyledons better than small-sized acorns. These results showed that most cotyledons are not necessary for seedling development, further supporting previous studies that show that detached radicles of various white oak species exhibit the capacity to produce normal seedlings even when cotyledons are pruned during the very early stages of seedling development^28^,^34^. In our study, we were unable to rule out the possibility of competition for resources among seedlings in each container. However, the effects of competition on seedling performance are expected to be minor because seedlings in each container survived regardless of the percentage of cotyledon removed in our study.

### Role of acorn size in countering cotyledon damage

In our study, partial removal of cotyledons showed minor influence on acorn survival and seedling development. In this context, the question that naturally incurs is why oaks produce acorns with extra cotyledon reserves. It has been widely accepted that seed size plays an important role at the stage of pre-dispersal predation caused by animals^12^,^23^. For example, large acorns appear to tolerate insect predators better than oak species with small acorn size^12^,^14^,^23^,^25^. Partial consumption of acorns by animals is very common in oaks^27^,^34^,^35^,^37^ and often explained by the satiation effect at seed level^23^,^38^,^39^. Giertych and Suszka^32^ showed a tolerance of partial damage (even up to 2/3) of *Q. robur* acorns to increasing cotyledon reduction. Armstrong and Westoby^29^ demonstrated that seedlings from large seeds tolerate defoliation better than those from small seeds, implying the role of seed size in seedling establishment in unfavorable conditions^7^. Moreover, the larger acorns of *Q. rugosa* and *Q. laurina* that were damaged during the seedling stage appeared to have better growth and survival rates than smaller ones^20^. Coupling with the fact that larger acorns have a higher germination rate^9^ and higher seedling fitness due to greater energy reserves^6^,^7^,^8^,^9^,^10^, we argue that oak production of large acorns aids in tolerating predation of animals^27^,^35^, and possibly assists in reducing other environmental stresses as well^40^,^41^.

### Role of acorn size in facilitating seed dispersal by animals

Most plant species are zoochoric and rely on various herbivores (rodents and birds) to disperse seeds^1^,^11^,^42^,^43^. Acorn size plays an important role in determining caching and dispersal decisions of rodents and birds^1^,^43^,^44^. We propose that this extra investment of parental resources in large acorns can be used to facilitate better seed dispersion. Rodents often prefer larger acorns over smaller ones because of the higher energy content of larger seeds^45^, so that larger acorns appear to have a consistent dispersal advantage over small acorns^1^,^45^,^46^. Perea et al.^27^ further found that large acorns of are more likely to be partially damaged prior to being cached by rodents, while small ones are more likely to be consumed immediately instead of being partially eaten. Large acorns were often dispersed further from the parent trees, and were more likely to be cached and survive than small acorns^1^,^47^, reflecting the role of acorn size in determining seed fates^40^,^44^,^48^,^49^,^50^,^51^. Recent studies showed that seeds with larger sizes exhibited higher mutualism but low predation with seed dispersers^52^,^53^. In our study, large acorns were more likely to be dispersed and cached by small rodents, compared with small-sized acorns. Based on the effect of cotyledon removal on acorn survival and seedling performance, this suggests that acorns of *Q. mongolica*, and possibly other oak species, act more as food to attract potential dispersers for better dispersion rather than as energy reserves to support oak seedling development.

## Conclusions

Although acorn tolerance to cotyledon damage has been well reported in various oak species, e.g., *Q. rugosa* and *Q. laurina*^20^, *Q. suber*^25^, *Quercus crispula*^54^, *Q. mongolica*^13^,^55^, *Q. variabilis*^14^, *Q. aliena*^23^, *Q. robur*^17^,^32^, *Q. pyrenaica*^27^, *Q. coccifera*^37^, and *Q. aliena var. acuteserrata*^38^, our study is one of the few showing the effects of successive cotyledon removal on acorn germination and seedling development. Results of our current experiment and data from the literature show that cotyledon reserves are not essential for acorn germination and seedling survival, suggesting that 50% (or more) of cotyledons function to counter seed predators. Acorns of larger sizes not only tolerate more damage but show advantages in the seed dispersal processes, demonstrating another important mechanism by which oak species ensure their dispersal. Producing large acorns may be more important for oaks to manipulate seed predators and dispersers than to provide seedlings with energy reserves.

## Author Contributions

X.F.Y. conceived and designed the experiments. C.Q.L., G.Q.L. and M.M.Z. performed the experiments. C.Q.L. and Z.Y.W. analyzed the data and prepared all figures. X.F.Y. and Z.Y.W. wrote the main manuscript. All authors reviewed the manuscript.

## Figures and Tables

**Table 1:** The coefficients, P and R^2^ values in linear regression model using seedling height, leaf number and seedling mass as the responses and initial seed mass, post-cut mass, and percent cotyledon loss as predictors

Responses	Predictors	Coefficient	P value	R^2^
Seedling height	Initial mass	0.043	0.781	0.002
	Post-cut mass	0.337	<0.001*	0.113
	Percent cotyledon loss	−0.402	<0.001*	0.161
Leaf number	Initial mass	0.013	0.932	0.000
	Post-cut mass	0.249	<0.001*	0.062
	Percent cotyledon loss	−0.242	<0.001*	0.059
Seedling mass	Initial mass	0.225	0.142	0.051
	Post-cut mass	0.149	<0.001*	0.386
	Percent cotyledon loss	−0.354	<0.001*	0.126
